# The Relationship Between Symptom Experiences and Quality of Life in Patients on Hemodialysis: The Mediating Role of Positive Psychological Capital

**DOI:** 10.3390/healthcare14203420

**Published:** 2026-10-13

**Authors:** Seung Won Ryu, Hye Young Kim, Won Hee Jun

**Affiliations:** 1 Dongsan Medical Center, College of Nursing, Keimyung University, Daegu 42601, Republic of Korea; abby9091@naver.com; 2 College of Nursing, Keimyung University, Daegu 42601, Republic of Korea; hye11533@kmu.ac.kr

**Keywords:** hemodialysis, nursing, psychological capital, quality of life, symptom assessment

## Abstract

**Highlights:**

**What are the main findings?**

• Positive psychological capital partially mediated the relationship between symptom experience and quality of life among patients on hemodialysis.
• Improving the quality of life of patients on hemodialysis requires not only reducing symptom experience but also strengthening their positive psychological capital.

**What are the implications of the main findings?**

• Nurses working in dialysis units should conduct comprehensive patient assessments by introducing screening tools that evaluate both symptom experience and levels of positive psychological capital among patients on hemodialysis.
• Nurses can provide various activities, educational materials, and psychological support to foster positive psychological capital for patients on hemodialysis.

**Abstract:**

**Background:** The quality of life of patients on hemodialysis is a critical indicator of their prognosis and mortality, making its enhancement particularly important. **Objective:** This study aimed to examine the mediating effect of positive psychological capital on the relationship between symptom experience and quality of life in patients on hemodialysis. **Methods:** This study employed a cross-sectional descriptive design. The participants were 156 patients on hemodialysis. Data were collected using structured self-report questionnaires. Mediation analysis was performed using the SPSS PROCESS Macro (Model 4), and the significance of the indirect effect was evaluated via bootstrapping. **Results:** The mean quality of life score was 3.17 out of 5. The quality of life of participants significantly differed by employment status. Positive psychological capital significantly mediated the relationship between symptom experience and quality of life (B = −0.18, SE = 0.06, 95% CI [−0.30, −0.07]). **Conclusions:** The findings of this study suggest that mitigating symptom experiences and fostering positive psychological capital may help improve the quality of life in patients on hemodialysis.

## 1. Introduction

Chronic kidney failure has the second highest incidence rate in the world, with 18.8 cases per one million people. The number of patients with chronic kidney failure in the Republic of Korea increased from 127,068 in 2022 to 161,639 in 2024, showing a steady annual increase. [1,2]. Hemodialysis is a renal replacement therapy used to sustain the lives of patients with chronic kidney failure; 83.7% of patients with chronic kidney failure in Korea receive hemodialysis, making it the most commonly used form of renal replacement therapy [2,3].

Hemodialysis is a treatment in which blood is regularly circulated outside the body through a dialysis machine two to three times per week for approximately four hours per session to remove waste products and excess fluid before being returned to the body [4]. Although hemodialysis is an effective and stable renal replacement therapy demonstrating relatively superior 5-year survival rates compared to peritoneal dialysis [5], patients on hemodialysis frequently encounter various physical and emotional challenges, time constraints, and changes in social roles, which contribute to a significantly lower quality of life than that of the general population [6,7].

Quality of life in patients on hemodialysis is a major indicator that may affect prognosis and mortality, and poor quality of life may increase hospitalization and mortality rates [8]. The marked disparity in quality of life scores between hemodialysis survivors and non-survivors underscores the importance of this metric in clinical assessments [8]. Therefore, assessing the quality of life of patients on hemodialysis and identifying related factors is imperative for developing strategies to improve their quality of life.

Symptom experience—the presence and severity of physical and emotional symptoms experienced by patients—has been consistently emphasized as a major factor associated with quality of life among patients on hemodialysis [9,10,11]. Patients undergoing hemodialysis predominantly experience concurrent physical and psychological symptoms, which have been shown to cause difficulties across various domains of daily life [9,12,13]. Among the physical symptoms experienced by these patients, fatigue and impaired mobility reduce the ability to perform activities of daily living, thereby negatively affecting quality of life, while pruritus decreases quality of life through depressed mood and deterioration in sleep quality [14,15]. Among emotional symptoms, depression may negatively affect quality of life through demoralization [16].

The symptom experience of patients on hemodialysis may not only directly reduce their quality of life but also be indirectly associated with quality of life through mediating factors [11]. Therefore, identifying these factors is crucial for broadening the understanding of quality of life among patients on hemodialysis and for establishing specific intervention strategies.

Previous studies have confirmed that patients on hemodialysis experience more symptoms, such as constipation, chest pain, and sleep disturbance, perceive greater discomfort and have lower self-management ability, resulting in lower self-efficacy [17]. In addition, physical symptoms, such as fatigue, headache, muscle cramps, dyspnea, and dizziness, due to hemodialysis treatment make it difficult for them to maintain their previous daily activities, including school life, housework, and social activities [6,7]. Consequently, they may lose hope or have difficulty maintaining optimism [18]. Therefore, symptom experience and positive psychological resources are considered to be closely related.

Meanwhile, positive psychological capital plays a key role in regulating the persistent stress resulting from the disease itself and the external environment and may contribute to improving quality of life by helping individuals evaluate stressful events more positively [19]. Positive psychological capital may serve as an important psychological resource that facilitates adaptive coping, self-management behaviors, and treatment adherence, which may contribute to better quality of life among patients with chronic diseases [20,21]. Turliuc and Candel [22] noted that individuals with high positive psychological capital adapt well to change and show greater confidence in their abilities, allowing them to overcome stress more effectively, which in turn can help improve their quality of life. Furthermore, a previous study has shown that resilience, one of the subdimensions of positive psychological capital, acts as a mediating factor in the relationship between symptom experience and quality of life in patients on hemodialysis [11].

Patients on hemodialysis tend to experience demoralization and easily lose hope and optimism because they must adhere to a lifelong treatment regimen [23,24]. In addition, successful self-care behaviors, such as dietary control, fluid restriction, and medication adherence, are important during the course of hemodialysis treatment, and resilience and self-efficacy may play important roles in achieving these behaviors [25,26]. Given that the characteristics of patients on hemodialysis are associated with various positive psychological resources, positive psychological capital, a multidimensional and integrated positive psychological state comprising self-efficacy, resilience, hope, and optimism [27], may be considered a potential mediating factor in the association between symptom experience and quality of life among patients on hemodialysis, as it serves as a psychological resource that facilitates adaptation to chronic illness and promotes self-care behaviors for symptom management.

Taken together, the results of previous studies indicate a potential link, suggesting that patients’ symptom experience may not only directly relate to their quality of life but could also be associated with it indirectly through psychological resources, such as positive psychological capital. However, previous studies have primarily focused on specific components of positive psychological capital [11,28]. To date, limited evidence has examined positive psychological capital as an integrated, multidimensional construct encompassing self-efficacy, resilience, hope, and optimism, particularly its potential mediating role in the association between symptom experience and quality of life among patients on hemodialysis [11,27,28]. Therefore, this study is intended to address this specific gap by examining the mediating role of positive psychological capital as an integrated psychological resource in the relationship between symptom experience and quality of life in patients on hemodialysis to provide basic data for the development of nursing interventions to improve their quality of life.

### Aims

The current study aimed to (1) identify the demographic characteristics, symptom experiences, positive psychological capital, and quality of life of the participants; (2) examine differences in quality of life according to the demographic characteristics of the participants; (3) investigate the correlations among symptom experiences, positive psychological capital, and quality of life in the participants; and (4) determine the mediating role of positive psychological capital in the relationship between symptom experiences and quality of life among the participants.

## 2. Materials and Methods

### 2.1. The Study Design and Participants

This study employed a cross-sectional descriptive correlational design and recruited participants using convenience sampling from 156 patients on hemodialysis at the hemodialysis unit of a tertiary hospital located in D city, Republic of Korea.

The inclusion criteria were patients diagnosed with chronic kidney failure and receiving hemodialysis 2–3 times weekly for over 3 months and those who were able to communicate verbally, and understand the contents of the questionnaire. The exclusion criteria were patients with acute kidney failure, those who had a kidney transplant, and those with a self-reported history of a mental disorder diagnosed according to the Diagnostic and Statistical Manual of Mental Disorders, Fifth Edition, Text Revision.

In this study, patients who had been on hemodialysis for at least 3 months were selected as subjects, based on the criteria suggested in the previous literature [29], in order to exclude temporary dialysis patients and include maintenance patients on hemodialysis who had adapted to dialysis treatment and were in a relatively stable condition.

The required sample size was calculated using the G*Power 3.1.9.7 program. For multiple regression analysis, with a significance level of 0.05, a medium effect size of 0.15, a statistical power of 0.90, and 9 predictor variables (7 demographic characteristics, symptom experience, and psychological capital), the minimum required sample size was 141 participants. The sample size of this study satisfied the required minimum sample size.

### 2.2. Measurements

#### 2.2.1. Symptom Experience

In this study, symptom experience was measured using the Dialysis Symptom Index, (DSI) developed by Weisbord et al. [30] and translated into Korean by Lim [31]. This 30-item instrument assesses the presence and severity of symptoms experienced by patients on hemodialysis during the previous week and comprises two subdomains: physical symptoms (21 items) and emotional symptoms (9 items). In the Korean version used in Lim [31], each symptom was scored from 0 to 5, with 0 indicating that the symptom was not present. For symptoms that were present, the degree of bothersomeness was rated on a 5-point Likert scale ranging from 1 (“not at all bothersome”) to 5 (“bothers very much”). In the study by Lim [31], Cronbach’s α = 0.90, and in the present study, Cronbach’s α was 0.91, demonstrating high internal consistency.

#### 2.2.2. Positive Psychological Capital

In this study, positive psychological capital was measured using the Korean version of the Positive Psychological Capital Questionnaire developed by Luthans et al. [27] and translated into Korean by Lim [32]. The instrument consists of 18 items across four subdomains: self-efficacy (5 items), optimism (5 items), hope (5 items), and resilience (3 items). Each item is rated on a 5-point Likert scale ranging from 1 (“strongly disagree”) to 5 (“strongly agree”), with higher scores indicating higher levels of psychological capital. In the study by Lim [32], Cronbach’s α = 0.92, and in the present study, Cronbach’s α was = 0.92, indicating excellent reliability.

#### 2.2.3. Quality of Life

In this study, quality of life was measured using the Korean version of the World Health Organization Quality of Life-BREF (WHOQOL-BREF) by Min et al. [33], originally developed by the WHO [34]. The instrument consists of 26 items, including two independent items assessing overall quality of life and general health, as well as four domains: physical health (7 items), psychological health (6 items), social relationships (3 items), and environment (8 items).

In this study, an overall quality of life score was calculated as the mean of the 24 items across the four domains of the WHOQOL-BREF: physical health (7 items), psychological health (6 items), social relationships (3 items), and environment (8 items). The two general items assessing overall quality of life and general health were excluded from this calculation. Before calculating the mean score, the three negatively phrased items were reverse-coded. The scores of the 24 items were then summed and divided by 24, yielding an overall mean score ranging from 1 to 5 [33]. Each item is rated on a 5-point Likert scale ranging from 1 (“not at all,” “very poor,” or “very dissatisfied”) to 5 (“completely,” “very good,” or “very satisfied”), with higher scores indicating better quality of life. Cronbach’s α was 0.89 in the study by Min et al. [33], and 0.92 in the present study.

### 2.3. Data Collection

Data were collected from 14 February to 14 March 2026, within the hemodialysis unit of a tertiary hospital situated in D city. Institutional approval was secured beforehand following detailed briefings with the head nurse and nephrologist regarding the study’s objectives and procedures. Potential participants undergoing hemodialysis were directly informed about the research aims, protocol, ethical safeguards, and anonymity assurance, and written informed consent was voluntarily obtained prior to enrollment. Questionnaires were completed within the unit’s education room and retrieved immediately by the researcher upon completion. Of the 159 distributed questionnaires, all were returned, and 156 valid responses—excluding three questionnaires with more than 10% unanswered questions—were ultimately utilized for the statistical analysis.

### 2.4. Ethical Considerations

Approval for this research was granted by the Institutional Review Board of Keimyung University Dongsan Hospital (DSMC 2026-01-044-002), and all protocols were strictly followed throughout the study. Prior to enrollment, potential participants received a comprehensive overview of the research aims and procedures from the investigator. Written consent was voluntarily provided by all individuals who agreed to take part. Participants were explicitly advised that their information would be used exclusively for research objectives, that their confidentiality and anonymity would be thoroughly protected, and that they retained the right to withdraw at any stage without incurring any penalty. For data protection, all records were kept in a secured, locked storage facility managed solely by the researcher for a three-year retention period, following which they will be permanently destroyed. Upon completion of the survey, participants were given a small token of gratitude valued at roughly 5000 KRW.

### 2.5. Data Analysis

All statistical analyses were performed using SPSS version 29.0 (IBM Corp., Armonk, NY, USA) and PROCESS macro version 4.2. The participants’ demographic characteristics and primary variables were evaluated using descriptive statistics including frequencies, percentages, means, and standard deviations. Differences in quality of life according to demographic characteristics were examined via independent t-tests and ANOVA. The correlations between symptom experience, psychological capital, and quality of life were analyzed using Pearson’s correlation coefficients. To test the mediating effect of positive psychological capital on the relationship between symptom experience and quality of life, regression analyses were conducted following Baron and Kenny [35]’s three-step procedure. Specifically, Step 1 tested the direct path from the independent variable to the dependent variable, Step 2 examined the effect of the independent variable on the mediator, and Step 3 incorporated both the independent variable and mediator into the regression equation predicting the outcome. To ensure the validity of the regression model, the normality and homoscedasticity of the residuals were assessed using the Kolmogorov–Smirnov test and the Breusch–Pagan test, respectively. Multicollinearity among the independent variables was assessed using tolerance and the variance inflation factor (VIF). Furthermore, mediation analysis was performed using SPSS PROCESS Macro (Model 4) [36]. The demographic variables that showed significant differences in quality of life according to the independent t-test or ANOVA results were dummy-coded and included as covariates in the analysis. A bootstrapping technique with 5000 resamples was applied to test the significance of the indirect effect, with statistical significance determined using bias-corrected 95% confidence intervals.

## 3. Results

### 3.1. Differences in Quality of Life According to Participants’ Demographic Characteristics

Of the 156 participants, 80 (51.3%) were women and 76 (48.7%) were men. The largest age group was 60 – < 70 years (n = 52, 33.3%). More than half of the participants reported having no religious affiliation (n = 95, 60.9%). Most participants were living with their family (n = 123, 78.8%), and unemployed (n = 115, 73.7%). The most frequent duration of hemodialysis was five years or more (n = 84, 53.8%), with an overwhelming majority of patients receiving treatment three times weekly (n = 146, 93.6%; Table 1). The quality of life of participants significantly differed by employment status (t = 2.29, *p* = 0.028). Employed participants (mean = 3.37, SD = 0.59) had significantly higher quality of life scores than those who were unemployed (mean = 3.10, SD = 0.71; Table 1).

### 3.2. Levels of Symptom Experience, Psychological Capital, and Quality of Life

The mean score of symptom experience was 1.07 (SD = 0.71) out of 5. The mean subscale scores of symptom experience were 1.36 (SD = 1.05) for emotional symptoms and 0.95 (SD = 0.64) for physical symptoms. The mean score of positive psychological capital was 3.08 (SD = 0.96) out of 5. Among the positive psychological capital subscales, scores were highest for resilience (mean = 3.46, SD = 1.17), followed by optimism (mean = 3.25, SD = 1.10), self-efficacy (mean = 2.96, SD = 1.08), and hope (mean = 2.79, SD = 1.11). The mean score of quality of life was 3.17 (SD = 0.69) out of 5. The mean scores of the subscales were 3.44 (SD = 0.69), 3.22 (SD = 0.78), 3.02 (SD = 0.74), 3.00 (SD = 1.00), and 2.70 (SD = 0.91) for the environment, psychological, physical health, social relationships, overall quality of life and general health, respectively (Table 2).

### 3.3. Correlations Between Variables

The participants’ quality of life was significantly and negatively correlated with symptom experience (r = −0.42, *p* < 0.001) and positively correlated with positive psychological capital (r = 0.69, *p* < 0.001). In addition, symptom experience showed a significant negative correlation with positive psychological capital (r = −0.32, *p* < 0.001; Table 3).

### 3.4. Mediating Effect of Positive Psychological Capital in the Relationship Between Symptom Experience and Quality of Life

Following Baron and Kenny [35], a three-step regression analysis was conducted to verify the mediating role of positive psychological capital in the relationship between symptom experience and quality of life. To ensure the validity of the regression model, the normality and homoscedasticity of the residuals were assessed using the Kolmogorov–Smirnov test and the Breusch–Pagan test, respectively. The results confirmed that all assumptions were met, with the Kolmogorov–Smirnov test indicating a normal distribution of residuals and the Breusch–Pagan test showing no significant heteroscedasticity, with all *p*-values exceeding 0.05 (Table 4). In addition, multicollinearity among the independent variables was assessed. The VIF values ranged from 1.04 to 1.14, and the tolerance values ranged from 0.88 to 0.96, indicating no evidence of problematic multicollinearity among the predictors included in the final regression model. The demographic variables (employment status) that showed significant differences in quality of life according to the independent t-test results were used as independent variables and entered into the regression model as dummy variables.

In the first step, symptom experience (B = −0.39, *p* < 0.001) was a significant predictor of quality of life. In the second step, symptom experience was a significant predictor of positive psychological capital (B = −0.40, *p* < 0.001). In the third step, positive psychological capital was a predictor of quality of life when controlling for symptom experience and employment status (B = 0.44, *p* < 0.001). Furthermore, the effect of symptom experience on quality of life in this context was statistically significant but smaller (B = −0.21, *p* < 0.001) than that in the first step (B = −0.39, *p* < 0.001; Table 4).

To further verify the significance of the mediating role of positive psychological capital on the relationship between symptom experience and quality of life, employment status, which differed significantly in the dependent variable quality of life, was dummy-coded and controlled for. Bootstrapping analysis was further performed; the bias-corrected 95% confidence interval for the estimated indirect association through positive psychological capital ranged from −0.30 to −0.07, excluding zero. In other words, the indirect association through positive psychological capital was statistically significant in the relationship between symptom experience and quality of life (B = −0.18, SE = 0.06, 95% CI [−0.30, −0.07]; Table 4).

## 4. Discussion

This study identified the mediating role of positive psychological capital in the relationship between symptom experience and quality of life among patients on hemodialysis.

The results showed that the participants’ mean symptom experience score was 1.07 out of 5, with emotional symptom experiences rated higher than physical symptom experiences across the subdomains. These findings were consistent with those of Jeong and Yang [10], who investigated patients on hemodialysis using the same instrument as that used in the present study.

One reason for these findings may be that medical staff members’ interventions for patients on hemodialysis are primarily focused on physical conditions, such as measuring vital signs, administering medication, and assessing physical symptoms, giving less priority to emotional symptoms [37]. Jeong and Yang [10]’s results also suggested that nurses working in dialysis units should encourage patients to express their emotional symptoms. To this end, they need to enhance their empathetic communication skills when assessing patients’ symptom experiences in this patient group so that patients can express their emotional symptoms more comfortably.

In this study, the mean score for positive psychological capital was 3.08 out of 5, and among its subdimensions, hope scored the lowest. Patients on hemodialysis experience psychological difficulties attributable to dependence on dialysis, fear of the future, and anxiety about death, which may make it difficult for them to maintain hope [13,24]. Hope in this patient population can reduce suicidal ideation, alleviate anxiety, depression, and stress, enhance self-care performance, and positively influence patients’ ability to persist with treatment without giving up [24,38]. Furthermore, positive-thinking training can alleviate feelings of despair, reinforce positivity, and promote reality acceptance in patients undergoing hemodialysis, thereby enhancing their hope [39,40]. Thus, it is necessary to actively introduce and apply this intervention to this patient group.

In this study, participants scored moderately on quality of life (mean score of 3.17 out of 5.00), similar to the findings of Lim and Kwon [41], who reported a mean score of 3.02, using the same instrument in patients on hemodialysis. Patients on hemodialysis have been reported to have a low quality of life not only because long-term and continuous treatment imposes a substantial financial burden, but also because they frequently experience repeated hospitalizations, a high symptom burden, and various psychosocial difficulties, including depression, anxiety, and social isolation [42]. Although the quality of life of participants in the present study was moderate, given its close association with the prognosis and mortality of patients on hemodialysis [8], healthcare providers should pay continuous and active attention to improving the quality of life of these patients.

In terms of quality of life according to participants’ demographic characteristics, those who were employed reported higher quality of life than those who were not. These results are consistent with Lee and Park [43], who demonstrated that employed patients on hemodialysis had a greater ability to accept life positively. Given the observed association between employment status and quality of life, as well as the structural barriers to employment imposed by treatment-related time constraints [44], nurses need to be attentive to patients’ employment-related challenges and provide appropriate support based on their individual needs and preferences. However, this association should not be interpreted as evidence that employment-related support improves quality of life. A future longitudinal study is warranted to clarify the direction of this relationship for patients on hemodialysis.

The present study also demonstrated that positive psychological capital mediated the relationship between symptom experience and quality of life among patients on hemodialysis. Specifically, lower levels of symptom experience were associated with a higher quality of life, and this relationship was partially explained by higher levels of psychological capital. In other words, symptom experience was associated with patients’ quality of life directly as well as indirectly through psychological capital. To date, no previous study has specifically examined the mediating role of positive psychological capital in the relationship between symptom experience and quality of life among patients on hemodialysis, making a direct comparison with the present findings difficult. However, previous studies have reported the mediating role of the subdomains of positive psychological capital. For example, resilience was found to mediate the relationship between symptom experience and quality of life in patients on hemodialysis [11]. Similarly self-efficacy mediated the relationship between symptom experience and health-related quality of life in patients with chronic obstructive pulmonary disease [28], which is consistent with the findings of the present study.

A heavy symptom burden experienced by patients on hemodialysis often leads to feelings of despair, reduced optimism about the future, and uncertainty about their illness, which, in turn, diminish their confidence in self-care and can result in lower positive psychological capital [18,45]. In addition, patients with low positive psychological capital tend to have negative attitudes toward treatment and experience difficulty adhering to treatment plans, which may ultimately result in poorer quality of life [46]. Therefore, improving the quality of life of patients on hemodialysis requires not only reducing symptom experience but also strengthening their positive psychological capital.

To reduce symptom experience, nurses should provide systematic and repeated individualized health education to help patients effectively perform self-care behaviors, including dietary and fluid restrictions, adherence to prescribed medications, and vascular access management [9,23]. Telehealth-based cognitive behavioral therapy has been found to be effective in reducing fatigue, pain, and depression in patients undergoing long-term hemodialysis [47]. Educational videos provided during hemodialysis have also been shown to reduce symptom burden by improving patients’ understanding of their symptoms and teaching symptom management strategies [48]. Furthermore, a structured exercise program was reported to be an effective non-pharmacological intervention for reducing physical symptoms, including constipation, loss of appetite, restless legs, and fatigue, as well as psychological symptoms, including worry, anxiety, sleep disturbance, and sadness, among patients on hemodialysis [49]. Accordingly, nurses should regularly offer diverse educational resources, including psychological interventions, throughout the hemodialysis process to enhance symptom management. In addition, they should design practical home-based exercise programs, promote active patient engagement, and provide consistent monitoring and feedback.

Positive psychological capital can be enhanced through online self-directed learning, which teaches the concept of positive psychological capital and encourages individuals to develop specific plans to achieve their goals by utilizing their personal strengths [50]. Virtual reality-based positive psychological interventions that incorporate strategies such as noticing and amplifying positive events, gratitude, behavioral activation, mindfulness, and positive reappraisal have been proposed as a potential approach to promote positive emotions and psychological well-being among patients on hemodialysis [51]. Furthermore, the literature has suggested that nurse-led psychosocial interventions may support positive psychological resources in patients experiencing psychological distress [52]. Therefore, nurses need to develop online self-directed learning and virtual reality-based programs to enhance the positive psychological capital of patients on hemodialysis.

### 4.1. Limitations and Directions for Future Research

This study has some limitations. First, the data were collected using convenience sampling from a single region, which may have introduced selection bias and limited the generalizability of the findings to a broader population of hemodialysis patients. Thus, future studies incorporating diverse geographic regions and multiple institutions are warranted. Second, regarding the statistical power of the mediation model, while the sample size was deemed adequate for multiple regression analyses, dedicated power calculations specifically optimized for testing indirect effects via bootstrapping were not performed a priori. Although the robust bootstrap resampling procedure (5000 subsamples) was utilized to enhance estimation reliability, future studies with larger, more diverse samples are warranted to replicate and confirm these indirect associations. Third, this study was limited by the omission of various sociodemographic and clinical characteristics potentially associated with quality of life among patients on hemodialysis. In addition, covariates were selected primarily based on statistical significance in preliminary bivariate analyses rather than on a comprehensive consideration of clinically and theoretically relevant confounding factors. Consequently, important confounders may not have been adequately accounted for, and residual confounding cannot be excluded. Therefore, the findings from the adjusted mediation analysis should be interpreted with caution. Future studies should incorporate a broader range of sociodemographic and clinical characteristics, including educational level, income, serum albumin, serum phosphorus levels, and depression, and consider clinically and theoretically relevant factors when selecting covariates to more comprehensively examine the associations among the study variables. Fourth, there may be conceptual and measurement overlap among the key variables in this study. Therefore, future studies should examine discriminant validity among the variables using confirmatory factor analysis and conduct additional sensitivity analyses using only physical symptom-related items to assess the extent to which overlap among measurement items may have influenced the study findings. Fifth, because symptom experience, positive psychological capital, and quality of life were assessed using self-report questionnaires during the same study period, the possibility of common method variance cannot be excluded. This may have influenced the observed associations among the study variables. Future studies should consider using multiple assessment methods or collecting data at different time points to minimize potential common method bias. Sixth, given the cross-sectional design of this study, the temporal ordering and directionality of the associations among study variables could not be established. Although the present study examined a theoretically proposed model in which positive psychological capital was positioned between symptom experience and quality of life, plausible reverse or reciprocal relationships cannot be excluded. Therefore, the observed indirect association should not be interpreted as evidence of temporal or causal mediation. Future studies utilizing longitudinal designs—or ideally, randomized controlled trials—are recommended to better establish the causality among these variables.

### 4.2. Implications for Nursing Practice

This study provides insights into potential nursing care approaches to addressing quality of life among patients on hemodialysis. Clinical nurses should consider positive psychological capital alongside symptom experience when assessing factors associated with quality of life in these patients. In particular, nurses working in dialysis units may consider incorporating screening tools that assess both symptom experience and levels of positive psychological capital into comprehensive patient assessments. Based on our findings, interdisciplinary collaboration may be beneficial for identifying patients who may be vulnerable to lower quality of life and facilitating referrals to appropriate mental health professionals when needed. Furthermore, nurses may consider providing educational resources and psychological support that address patients’ psychological needs and positive psychological resources. However, given the cross-sectional design of this study, these recommendations should be regarded as potential approaches rather than interventions with established effectiveness. Future longitudinal and intervention studies are needed to clarify the relationships among symptom experience, positive psychological capital, and quality of life and to evaluate the effectiveness of nursing interventions targeting these factors.

## 5. Conclusions

The findings showed a statistically significant indirect association between symptom experience and quality of life through positive psychological capital. In other words, symptom experience was directly associated with quality of life, and indirectly associated through positive psychological capital. Positive psychological capital appears to be one of the psychological pathways through which symptom experience is associated with quality of life. Therefore, future studies should develop programs that enhance positive psychological capital in patients on hemodialysis who struggle with symptom experience, and evaluate their effects on quality of life.

## Figures and Tables

**Table 1:** Differences in quality of life according to participants’ demographic characteristics (N = 156).

Characteristics	Category	n (%)	Quality of Life ^ǂ^	
			Mean ± SD	t/F(p)
Gender	Men	76 (48.7)	3.06 ± 0.64	−1.94 (0.054)
	Women	80 (51.3)	3.27 ± 0.71	
Age (years)	40–< 50	17 (10.9)	3.17 ± 0.64	0.08 (0.973)
	50–< 60	39 (25.0)	3.16 ± 0.53	
	60–< 70	52 (33.3)	3.20 ± 0.68	
	≥70	48 (30.8)	3.16 ± 0.83	
Religion	Yes	61 (39.1)	3.28 ± 0.70	1.52 (0.130)
	No	95 (60.9)	3.10 ± 0.67	
Family members living	Yes	123 (78.8)	3.19 ± 0.65	−0.52 (0.604 )
together	No	33 (21.2)	3.12 ± 0.81	
Employment status	Employed	41 (26.3)	3.37 ± 0.59	2.22 (0.028 )
	Unemployed	115 (73.7)	3.10 ± 0.71	
Duration of	<1	18 (11.5)	3.08 ± 0.54	0.18 (0.911)
hemodialysis (years)	1–< 3	30 (19.3)	3.23 ± 0.69	
	3–< 5	24 (15.4)	3.15 ± 0.61	
	≥5	84 (53.8)	3.17 ± 0.74	
Frequency of dialysis per week	Twice	10 (6.4)	3.11 ± 0.51	−0.31 (0.759)
	Thrice	146 (93.6)	3.18 ± 0.70	

SD, standard deviation. ^ǂ^ Value excluding the two items assessing overall quality of life and general health.

**Table 2:** Levels of symptom experience, positive psychological capital, and quality of life (N = 156).

Variable	Minimum	Maximum	Mean ± SD
Symptom experience	0.00	3.11	1.07 ± 0.71
Emotional symptoms	0.00	4.22	1.36 ± 1.05
Physical symptoms	0.00	3.05	0.95 ± 0.64
Positive psychological capital	1.00	5.00	3.08 ± 0.96
Resilience	1.00	5.00	3.46 ± 1.17
Optimism	1.00	5.00	3.25 ± 1.10
Self-efficacy	1.00	5.00	2.96 ± 1.08
Hope	1.00	5.00	2.79 ± 1.11
Quality of life ^ǂ^	1.67	4.89	3.17 ± 0.69
Environment	1.63	4.88	3.44 ± 0.69
Psychological health	1.33	5.00	3.22 ± 0.78
Physical health	1.29	5.00	3.02 ± 0.74
Social relationships	1.00	5.00	3.00 ± 1.00
Overall quality of life and general health	1.00	5.00	2.70 ± 0.91

^ǂ^ Value excluding the two items assessing overall quality of life and general health.

**Table 3:** Correlations between Variables (N = 156).

Variable	Symptom Experience	Positive Psychological Capital	Quality of Life
		r(*p*)	
Symptom experience	1		
Positive psychological capital	−0.32 (<0.001)	1	
Quality of life	−0.42 (<0.001)	0.69 (<0.001)	1

**Table 4:** Mediating Effect of Positive Psychological Capital in the Relationship between Symptom Experience and Quality of Life (N = 156).

Variable	Step 1				Step 2				Step 3				95% CI
	Y				M				Y				
	B	SE	β	*p*	B	SE	β	*p*	B	SE	β	*p*	LLCI to ULCI
Employment status (Employed) ^†^	0.20	0.11	0.13	0.079	0.35	0.17	0.16	0.040	0.05	0.09	0.03	0.578	−0.13 to 0.23
X	−0.39	0.07	−0.40	<0.001	−0.40	0.10	−0.30	<0.001	−0.21	0.06	−0.22	<0.001	−0.33 to −0.10
M									0.44	0.04	0.62	<0.001	0.35 to 0.52
F (p)	18.11 (<0.001)				10.78 (<0.001)				55.66 (<0.001)				
R^2^ (Adjusted R^2^)	0.191 (0.181)				0.123 (0.112)				0.523 (0.514)				
Z (p) ^**^	0.71 (0.691)				0.41 (0.996)				0.46 (0.982)				
χ^2^ (p) ^††^	1.53 (0.466)				2.95 (0.229)				3.30 (0.347)				
Type of effect	B			SE			95% CI						
							LLCI				ULCI		
Total effect	−0.39			0.07			−0.53				−0.25		
Direct effect	−0.21			0.06			−0.33				−0.10		
Indirect effect	−0.18			0.06			−0.30				−0.07		

^†^ Dummy variables (reference: Unemployed), X = Symptom experience, M = Positive psychological capital, Y = Quality of life. LLCI = Lower Limit Confidence Interval, ULCI = Upper Limit Confidence Interval. ^**^ Kolmogorov–Smirnov normality test with standardized residual. ^††^ Breusch–Pagan homoscedasticity test with residuals. Indirect effects were tested with 95% bias-corrected bootstrap confidence intervals based on 5000 resamples.

## Data Availability

The data presented in this study are available on reasonable request from the corresponding author due to privacy and ethical restrictions.
